# International Centers of Excellence for Malaria Research

**DOI:** 10.4269/ajtmh.15-0407

**Published:** 2015-09-02

**Authors:** Malla R. Rao

**Affiliations:** Division of Microbiology and Infectious Diseases, National Institute of Allergy and Infectious Diseases, National Institutes of Health, Bethesda, Maryland

The past two decades have witnessed an extraordinary commitment to malaria control and elimination, which has been accompanied by a considerable reduction in global malaria morbidity and mortality. Although malaria research efforts have dramatically increased over this period,^1^ most clinical research projects still tend to be narrowly focused in scientific scope, and are conducted in a limited catchment area with a relatively homogeneous “at risk” population. In 2010, the National Institute of Allergy and Infectious Diseases established 10 International Centers of Excellence for Malaria Research (ICEMR) to support multidisciplinary research in diverse epidemiologic settings which span representative malaria-endemic regions around the world. Table 1 describes the locations of field sites and research focuses of each ICEMR. Research projects were designed to capture and track changes in the complex interactions between the human host, malaria parasite, and mosquito vector in different eco-epidemiologic settings. Unlike typical monitoring and evaluation programs, the ICEMRs are mandated both to adopt an integrated approach to malaria research, and to apply molecular epidemiology and genomics to study transmission and disease in the context of dynamically changing in disease prevalence and incidence (Table 1).

Following the initial establishment of the ICEMRs, *Acta Tropica* published a special supplement issue focusing on the ICEMRs, titled “Tackling the Malaria ‘End Game’: Regional Needs and Challenges for Successful Malaria Elimination.”^2^,^3^ This supplement described each of the Centers, their research projects, and their scientific scope. The articles described how individual Centers planned to implement multidisciplinary research approaches in the context of local and regional control efforts to provide evidence-based findings which inform the future course of malaria control and elimination efforts. Taking advantage of the network structure of the ICEMRs, which encourages and facilitates collaboration between centers, the ICEMRs have shared protocols and technologies and have worked to harmonize outcome and predictor variable definitions. In this issue, the ICEMRs jointly present early findings organized by on broad themes rather than by results specific to individual centers. This approach highlights the value of a broadly focused program and the generalizability of the findings from the ICEMR network.

In this research supplement, the ICEMRs attempt to provide a comprehensive view of the interplay between control interventions and epidemiology, vector ecology, parasite diversity, insecticide and drug resistance, pathogenesis, diagnostic performance, molecular epidemiology, biosignatures of transmission and immunity, and urban malaria across different settings. The impact of human migration, man-made ecological modifications, and climate and vector behavior on malaria transmission is extremely complex to study and quantify. The multidisciplinary nature of the ICEMR program enables each center to study these interactions in a specific, well-defined setting, and to subsequently compare findings across centers for consistency and variability. Table 2 provides an overview of the transmission settings in which the ICEMRs are located as well as study questions and approaches.

Although the incidence of malaria continues to decline in many parts of the world, some sites have experienced little or no change, and a few locations have seen incidence rise. The most pressing scientific questions will differ depending on the transmission setting. For example, when transmission is low, how is it sustained and what control and surveillance measures will be most effective at driving rates toward elimination? Where transmission levels remain high despite interventions,^4^ what are the factors or knowledge gaps that impede impact? Where malaria has recently dropped to low levels, what are the early warning signs of an impending epidemic? In addition to insecticide-impregnated bed nets and indoor residual spraying of insecticides, a number of novel control and prevention strategies have been proposed in recent years. Seasonal malaria chemoprophylaxis, mass drug administration, school-based treatment programs, targeted parasite elimination, and outdoor vector control are examples of a few approaches being tried in different settings.^5^–^7^ In addition to the direct impact these interventions might have on disease and infection rates, it is critical to study their effects on the immune status of the population, the genetic diversity of circulating parasite strains, the evolutionary response of emerging and surviving strains, as well as impacts on the mix of vector species and their vectorial capacity. All of these factors will ideally be taken into account to maximize the likelihood of achieving malaria elimination as quickly as possible. As a network of multidisciplinary research centers in diverse epidemiological settings, the ICEMRs are poised to provide insightful research findings that will inform the design and implementation of optimal malaria control and elimination programs.

## Figures and Tables

**Table 1:** ICEMR: description of centers

ICEMR	Locations/(sites)	Title	Grantee institution	Research focuses
Latin America	Colombia (Tierralta, Buenaventura, Tumaco, Quibdó) Peru (Sullana) Ecuador (Esmeraldas) Guatemala (Zacapa, Alta Verapaz, Escuintla)	Latin American Center for Malaria Research and Control	Caucaseco Scientific Research Center, Cali	-Malaria in low transmission settings
				•Role of asymptomatics in transmission and assessment of new tools for elimination
				•Ecological diversity of vectors and parasites
				•Clinical profile of complicated and uncomplicated malaria
Amazonia	Peru (Loreto) Brazil (Remansinho, Acrelândia)	Peruvian/Brazilian Amazon Center of Excellence in Malaria	University of California, San Diego	-Biology of hypoendemic malaria
				•Asymptomatic human reservoirs, new seroepidemiological tools
				•Ecology and environment related to malaria entomology
				•Immunology of transmission to mosquitoes
West Africa	Gambia (Gambissara) Senegal (Dakar) Mali (Dangassa, Dioro)	Population-Based Approach to Malaria Research and Control	Tulane University of Louisiana	-Impact of different control mix
				•Immune responses
				•Drug resistance
				•Insecticide resistance
Southern Africa	Zambia (Nchelenge, Choma) Zimbabwe (Mutasa)	Malaria Transmission and the Impact of Control Efforts in southern Africa	Johns Hopkins Bloomberg School of Public Health	-Factors influencing transmission and control in regions with:
				•Low-level endemicity approaching elimination
				•Resurgence after successful control
				•High transmission despite adequate control
East Africa	Uganda (Jinja, Kanungu, Tororo)	Program for Resistance, Immunology, Surveillance and Modeling of Malaria In Uganda	University of California, San Francisco	-Transmission at sites with varying intensities
				•Surveillance strategies
				•Sero-epidemiology
				-Drug and insecticide resistance
Malawi	Blantyre, Chikwawa, Thyolo	Determinants of Malaria Disease in Malawi	Michigan State University	-Monitoring impact in diverse geographic settings
				•Disease pathogenesis in severe malaria
				•School-based interventions
				-Urban Malaria
South Asia	India (Goa, Wardha, Dibrugarh, Ranchi, Mumbai)	Malaria Evolution in South Asia	University of Washington	-Parasite evolution in diverse transmission settings
				•Genome plasticity and drug resistance
				•Pathogenesis mechanisms
				•Innate human and vector protection
India	Chennai, Rourkela, Nadiad	Center for the Study of Complex Malaria In India	New York University	-Disease outcomes based on complexity of infection due to:
				•Mixed species
				•Multiple genotypes
				•Varied ecologies (urban vs. forest)
				•Different vectors
Southeast Asia	China (Ying Jiang) Thailand (Tha Song Yang) Myanmar (Kachin State)	Southeast Asia Malaria Research Center	Pennsylvania State UniversityUniversity Park	-Influence of cross-border migration on
				•Epidemics of malaria
				•Complexity of circulating genotypes
				-Evolution of drug resistance
				-Diagnostics for drug quality
Southwest Pacific	PNG (East Sepik, Madang) Solomon Islands (Central and Western Provinces)	Research to Control and Eliminate Malaria in southeast Asia and southwest Pacific	Case Western Reserve University	-Impact of transmission reduction on
				•Holoendemic malaria in mainland PNG
				•Hypoendemic malaria in Solomon Islands
				•Relative impact on *Plasmodium vivax* vs. *Plasmodium falciparum*

**Table 2:** ICEMR: description of centers: research settings and questions

Transmission setting	Perennial high level	Seasonal high levels	Unpredictable epidemics/re-emerging malaria	Urban, mining, deforested, border, and remote areas	Low-level approaching elimination
Study questions	•Reasons for failure and success of control measures	•Vector breeding sites and reservoirs	•Contribution of immune status	•Features of environmental modification	•Optimal surveillance strategies and diagnosis tools
	•New alternatives	•Changes in immunity and severity	•Changes in age and severity spectrum	•Detection of breeding sites	•Optimal control strategies and mix
			•Factors responsible for failure of control	•Contribution of migrants and human movement	
Outcomes and measures	•Species and strain diversity	•Vector behavior and mix	•Human movement and migration	•New environments for vector breeding sites	•Vector density
	•Insecticide resistance				
	•Drug resistance	•Immune status	•Introduction of novel strains	•Risk behavior of populations	•Serologic markers
	•Genetic factors of host	•Asymptomatic reservoirs	•Strain diversity and mix	•Vector detection and collection	•Landscape genetics
	•Asymptomatic reservoirs	•Gametocyte carriage	•Treatments, regimens, and control strategies	•Socioeconomic status and malaria risk	•Submicroscopic reservoirs
	•Immune markers of exposure and protection				
ICEMRs	West Africa, southern Africa, East Africa, Malawi, south Asia, India, southwest Pacific	Malawi, south Asia, India, southeast Asia	Latin America, Amazonia, southern Africa, East Africa, south Asia, southeast Asia	Latin America, Amazonia, West Africa, East Africa, Malawi, South Asia, India, southeast Asia	Latin America, Amazonia, southern Africa, India, southwest Pacific
Sites	Dangassa (Mali), Nchelenge (Zambia), Tororo and Kanungu (Uganda), Chikhwawa (Malawi), Dibrugarh and Rourkela (India), East Sepik and Madang (PNG)	Thyolo (Malawi), Ranchi and Rourkela (India), Kachin State (Myanmar)	Tumaco (Colombia), Esmeraldas (Ecuador), Loreto (Peru), Mutasa (Zimbabwe), Jinja (Uganda), Goa (India), Kachin State (Myanmar)	Quibdó (Colombia), Loreto (Peru), Dakar (Senegal), Jinja (Uganda), Blantyre (Malawi), Goa, Diburgarh and Chennai (India), Thai–Myanmar border, China–Myanmar border	Alta Verapaz (Guatemala), Buenaventura (Colombia), Sullana (Peru), Choma (Zambia), Nadiad (India), Central and Western Province (Solomon Islands)

ICEMR = International Centers of Excellence for Malaria Research.
